# Life-extended glycosylated IL-2 promotes Treg induction and suppression of autoimmunity

**DOI:** 10.1038/s41598-021-87102-4

**Published:** 2021-04-07

**Authors:** Aner Ottolenghi, Priyanka Bolel, Rhitajit Sarkar, Yariv Greenshpan, Muhammed Iraqi, Susmita Ghosh, Baisali Bhattacharya, Zoe V. Taylor, Kiran Kundu, Olga Radinsky, Roi Gazit, David Stepensky, Ron N. Apte, Elena Voronov, Angel Porgador

**Affiliations:** 1 The Shraga Segal Department of Microbiology, Immunology, and Genetics, Faculty of Health Sciences, Ben-Gurion University of the Negev, 84105 Beer Sheva, Israel; 2 National Institute for Biotechnology in the Negev, Ben-Gurion University of the Negev, 84105 Beer Sheva, Israel; 3 Department of Clinical Biochemistry and Pharmacology, Faculty of Health Sciences, Ben-Gurion University of the Negev, 84105 Beer Sheva, Israel

**Keywords:** Immunology, Applied immunology, Autoimmunity, Cytokines, Immunological disorders, Immunotherapy, Inflammation, Lymphocytes, Mucosal immunology

## Abstract

IL-2 is the master-regulator cytokine for T cell dependent responses and is crucial for proliferation and survival of T cells. However, IL-2-based treatments remained marginal, in part due to short half-life. Thus, we aimed to extend IL-2 half-life by flanking the IL-2 core with sequences derived from the extensively glycosylated hinge region of the NCR2 receptor. We termed this modified IL-2: “S2A”. Importantly, S2A blood half-life was extended 14-fold compared to the clinical grade IL-2, Proleukin. Low doses inoculation of S2A significantly enhanced induction of Tregs (CD4^+^ Regulatory T cells) in vivo, as compared to Proleukin, while both S2A and Proleukin induced low levels of CD8^+^ T cells. In a B16 metastatic melanoma model, S2A treatment was unable to reduce the metastatic capacity of B16 melanoma, while enhancing induction and recruitment of Tregs, compared to Proleukin. Conversely, in two autoimmune models, rheumatoid arthritis and DSS-induced colitis, S2A treatment significantly reduced the progression of disease compared to Proleukin. Our results suggest new avenues for generating long-acting IL-2 for long-standing treatment and a new technique for manipulating short-life proteins for clinical and research uses.

## Introduction

IL-2 is the master-regulator cytokine for T cells. It is a small soluble protein (at 15.5 kDa, 134AA), produced primarily by T cells and for T cells (although not exclusively). Discovered in 1975, it was first named T cell Growth Factor (TCGF) due to its T cell expansion properties^1^. This ability was the underlying cause of the introduction of this cytokine into the clinics as immunotherapy for cancer^2^. However, the short half-life of IL-2 in serum^3^, made such treatment a demanding process. Additionally, IL-2 had toxicity issues at higher doses needed for effective treatment, mostly manifesting as a vascular leaking syndrome (VLS) in the lungs^2^. Finally, the "IL-2 paradox" was recognized when patients were treated for a prolonged period with IL-2 often exhibited T cells anergy and cancer progression, rather than T cell activation and proliferation and consequently—cancer elimination. The subsequent discovery of the regulatory subset of T cells (Tregs), and their ability to downregulate immune-response, explained this apparent paradox^2^. These findings, along with new evolving trends in cancer therapy, were amongst the reasons IL-2 therapy fell out of grace. Currently, IL-2 is rarely used in the clinics and is only approved for metastatic melanoma and metastatic kidney cancer^2,4^.

Despite the clinical hurdle, research into IL-2 did not fade. Different approaches for modification of IL-2 were applied in order to generate long-acting IL-2 and to overcome the problems presented by treatment with the native protein. One method was the introduction of a single-point mutation that changed the binding properties to the different parts of the receptor, thus biasing the immune reaction towards the desired direction^5^. Another approach dealt with the short half-life problem by ligating the protein with stabilizing additions^6–9^. Other suggestions included using IL-2 as a treatment for autoimmune diseases, due to its Treg inducing capabilities^8,10^.

Natural cytotoxicity triggering receptor 2 (NCR2, NKp44, CD336) is a receptor unique to NK cells. It has two Ig like extracellular domains, and a highly glycosylated hinge region (mostly O-glycosylation)^11–16^. The richness of the O-glycans in the hinge region bears a resemblance to the O-glycosylation state of the C-terminal peptide (CTP) of hCGβ^17,18^. This observation encouraged us to investigate whether fragments of the hinge could mediate half-life elongation similarly to the data reported for the CTP of hCGβ^19^.

Elongation of half-life and changes of pharmacokinetic properties of long-acting IL-2 holds the key to making IL-2 treatment more "user-friendly," so it could be self-administered subcutaneously at the patient's home^20,21^. Additionally, the slow-release mechanism will result in long-lasting constant levels of IL-2 per administration and thus not only prolonging the intervals between injections but also potentially lowering the adverse side effects^5,21^. In the current study, we describe modified recombinant IL-2, which includes flanking sequences derived from the NCR2/NKp44 protein, called S2A. The S2A has a significantly extended half-life, and it acts as a long-acting IL-2 that primarily induces Treg activity that, in turn, suppresses autoimmunity.

## Results

### Recombinant IL-2 with flanking sequences derived from NKp44

**Figure 1:**
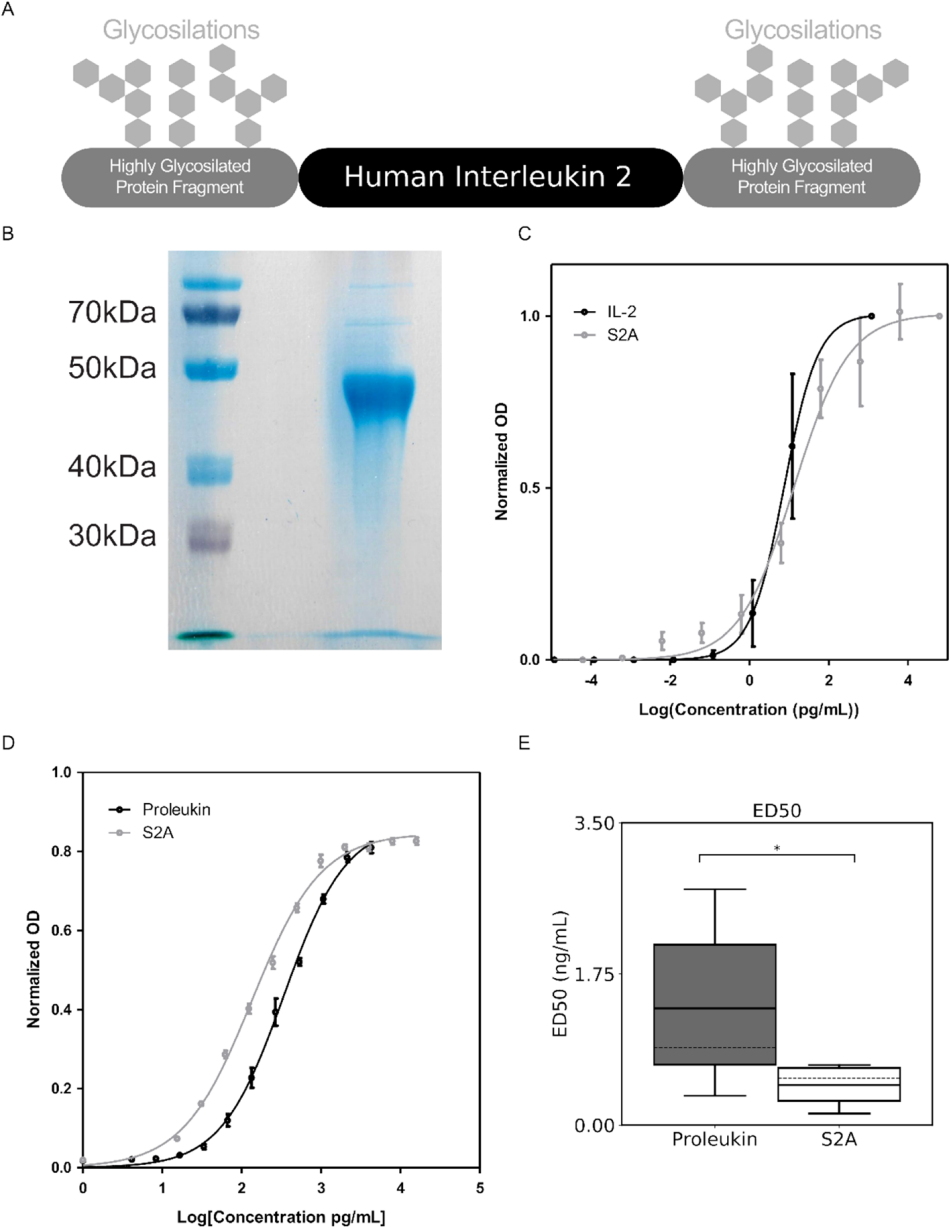
IL-2 variant design and in vitro functional analysis. (**A**) A scheme structure of the recombinant IL-2 S2A. The human IL-2 encoding sequence is flanked by two highly glycosylated fragments. S2A sequence also includes the IL-2 signal peptide at the N-terminal and His-tag sequence at the C-terminal. (**B**) S2A encoding plasmids were transfected into HEK293F cells, and protein was purified from media using the Ni-ion exchange column. Following purification, purity and size were determined by running eluted fraction on SDS-PAGE. (**C**) A representative ELISA binding curve (fitted to 4-parameter logistic) using IL-2 ELISA kit (Biolegend) (**D**) A representative CTLL-2 proliferation test, done using MTT assay (fitted to 4-parameter logistic. (**E**) Aggregated results of 5 independent CTLL-2 assays for different preparations of commercial IL-2 (Proleukin) and S2A. Mann–Whitney U test was used to determine significance (solid line—mean, crushed line—median; *, *P* < 0.05).

We previously defined amino acid sequences derived from the hinge region of NKp44 isoform 2 (NP_001186438.1) as capable of prolonging the biological half-life (T_½_) of human growth hormone (AP, unpublished results). These sequences included residues 130 to 163 (34AA; termed as SPA) and residues 164 to 199 (36AA; termed as APE) of NKp44 isoform 2^22^. Therefore, we aimed to test these sequences for proteins exhibiting a very short in vivo half-life, such as IL-2^3^. We produced recombinant human IL-2 as described in Fig. 1A in which human IL-2 (NP_000577.2, residues 21 to 153) is flanked by the SPA sequence and the APE sequence in the N- and C-termini, respectively (Fig. 1A). We rename this recombinant modified human IL-2 as “S2A”. The NKp44 hinge region is highly glycosylated, particularly with O-glycans^11–13^. The glycosylation of the SPA and the APE portion is preserved in the S2A, with the apparent molecular weight of nearly 50 kDa (Fig. 1B), which is in contrast to the calculated molecular weight of 25.2 kDa based on the AA sequence of the S2A^23^. We then aimed to test whether the SPA and APE modifications changed the structure and in vitro function of the IL-2 component of the S2A. We employed a commercial sandwich ELISA kit to measure the purified S2A and compared to recombinant human IL-2. The ELISA-based measured EC_50_ of the recombinant human IL-2 was 7.5 ng/ml, while that of S2A was 13.4 ng/ml (Fig. 1C and Table 1). The difference is within the experimental error range since the 95% confidence interval for S2A is 6.9 to 25.8 ng/ml. Alternatively, this difference could stem from the effect of glycosylation on the extinction coefficient or if SPA and APE additions affect the recognition by anti-IL-2 antibodies in this commercial sandwich ELISA^24^.

**Table 1:** Comparison of ELISA binding parameters.

	EC_50_ (ng/ml)	95% CI
IL-2 (Peprotech)	7.49	4.425–12.70
S2A	13.38	6.94–25.77

Convinced with the ELISA results, which suggest that the SPA and APE additions had not disturbed the structure of the IL-2, we further aimed to test the in vitro functional effect of this modification. The CTLL-2 cell proliferation assay is the standard assay to calculate the biological activity of IL-2, measured in IU/mg^25^. We compared S2A and the clinical-grade recombinant human IL-2, Proleukin. Based on the CTLL-2 assay, the measured ED_50_ of Proleukin was considerably lower than that of S2A (Fig. 1D, a representative result). This difference could not be attributed to experimental error range of the specific experiment. The measured average ED_50_ of S2A and Proleukin were 460 and 1350 pg/ml respectively, based on a summary of five independent experiments (Fig. 1E and Table 2): this represents a 2.9 fold change between S2A and Proleukin (*P*-value < 0.05), meaning that S2A has higher IUs per mg protein as compared to Proleukin. The manufacturer of Proleukin reported higher specific activity (IU/mg) than what we found in our assay; however, different measurements of specific activity for different Proleukin preparations, as compared to manufacturer data, was also reported by others^26^.

**Table 2:** Comparison of CTLL-2 proliferation assay parameters (n = 5).

	Mean ED_50_ (ng/ml)	95% CI
Proleukin	1.35	0.094–2.609
S2A	0.46	0.158–0.769

### Pharmacokinetic analysis of S2A and Proleukin

**Figure 2:**
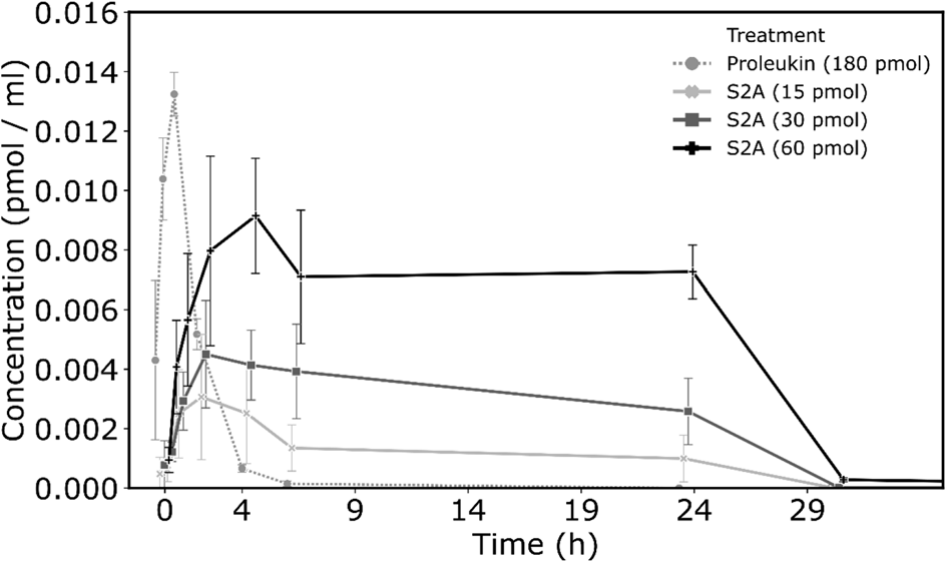
Blood concentrations of Proleukin and S2A following s.c. injection. Mice (n = 5) were injected subcutaneously with the indicated doses of Proleukin or S2A. Blood was then collected at 10 min, 30, min, 1 h, 2 h, 4 h, 24 h, 30 h, 42 h and 72 h. Levels of Proleukin or S2A were measured by ELISA. Bars represent SD.

Next, we wanted to examine S2A activity in vivo. To compare the PK of S2A and Proleukin following subcutaneous administration, mice (n = 5 per treatment group) were injected with S2A (15, 30, or 60 pmol) or with Proleukin (180 pmol). Peripheral Blood was sampled just prior to the protein injection as the time zero point and during the next 50 h. Figure 2 shows the PK curves where the area under the curve (AUC 0-t) is representing the bioavailability of the drug^27^. Our recombinant S2A gained 0.033, 0.093 and 0.194 pmol/ml × h for the three administration doses of S2A; compare to the AUC value for the Proleukin (0.02), although the inoculated dose was 180 pmol (8,000 IU) as compared to the 15, 30 and 60 pmol (3,000, 6,000 and 12,000 IU) S2A doses. Thus, the F_rel_ calculated for S2A is between 3.6 and 5.2 more than Proleukin (when calculated using the IU dosage, Table 3). In accordance with these observations, the clearance half-life (t_½_ clearance) of the S2A was near 10.5 h (14.1, 7.8, and 9.3 h were measured for the three doses) while the t_½_ clearance of Proleukin was significantly shorter (0.74 h, 44 min). In contrast to the sharp differences in values of AUC and t_½_ clearance, the Cmax values of S2A and Proleukin, representing the maximum drug concentration observed in the blood samples, manifest a similar pattern. Inoculation dose of 60 pmol (12,000 IU) S2A resulted in 0.009 pmol/ml (1.77 IU/ml) while administration dose of 180 pmol (8000 IU) Proleukin resulted in 0.0125 pmol/ml (0.99 IU/ml). Yet, the time to reach the Cmax (Tmax) was substantially shorter for the Proleukin (1 vs. 2.25 h for 8000 IU Proleukin and S2A, respectively), indicating that Proleukin is absorbed to the blood faster than the S2A (Fig. 2 and Table 3).

**Table 3:** The results of non-compartmental pharmacokinetic analysis of the Proleukin and S2A concentration versus time data.

	Proleukin 180 pmol (8000 IU)	S2A 15 pmol (3000 IU)	S2A 30 pmol (6000 IU)	S2A 60 pmol (12,000 IU)
t_½_ (h)	0.744 ± 0.07	14.1 ± 2.5	7.88 ± 2.8	9.3 ± 1.9
T_max_ (h)	1	1.75	3.2	1.8
C_max_ (pmol/ml)	0.0132 ± 0.0006	0.0031 ± 0.001	0.0047 ± 0.00135	0.0125 ± 0.001
AUC_0-t_ (pmol/ml × h)	0.246 ± 0.0006	0.0335 ± 0.01	0.0936 ± 0.03	0.194 ± 0.019
F_rel_ (IU fold)	1	3.64	5.08	5.27

Average ± SD, n = 5. Analysis performed in PKsolver add-in to Microsoft Excel.

### S2A is a potent inducer of Treg and less conducive to VLS

**Figure 3:**
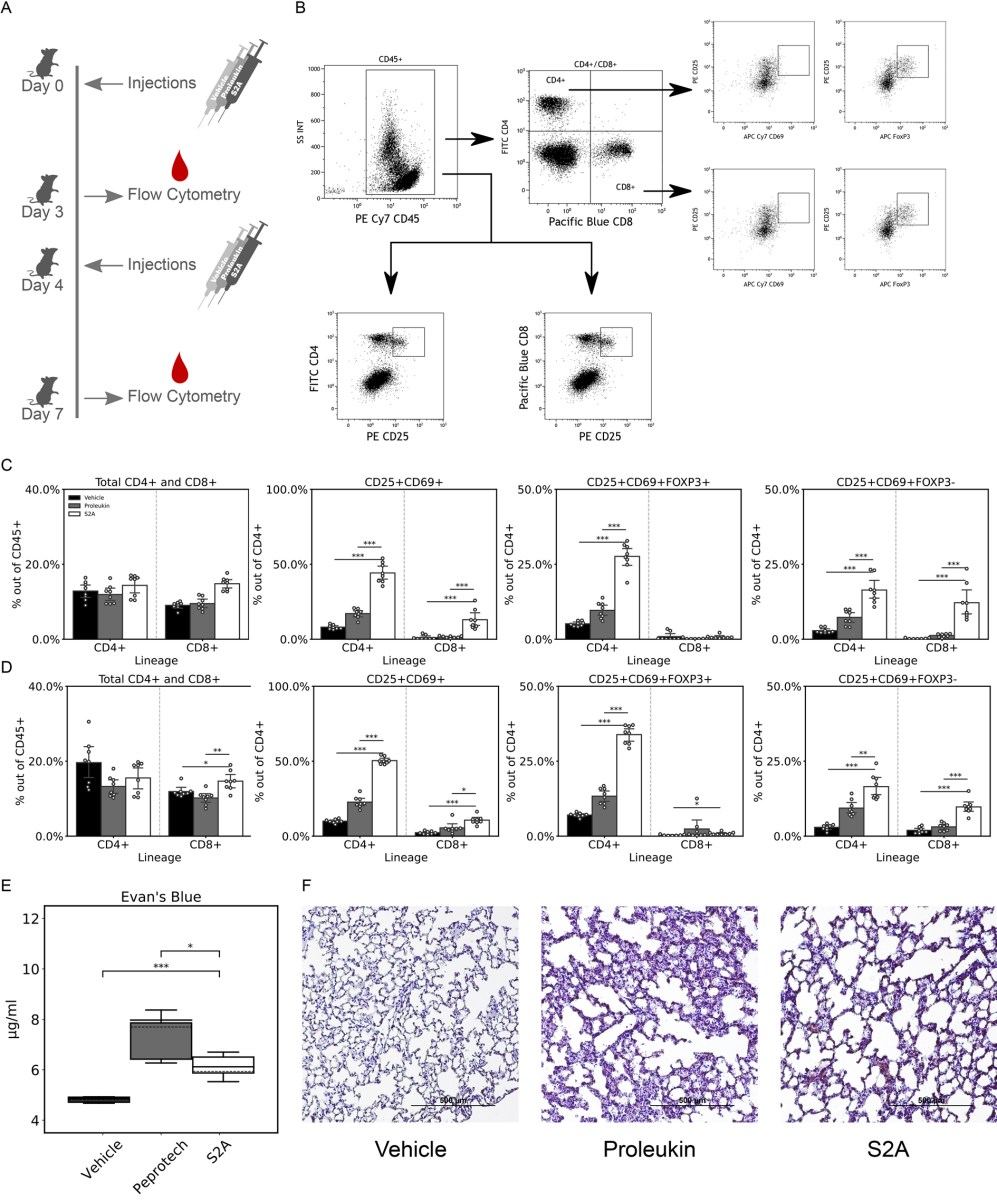
Induction of Treg by S2A inoculation. (**A**) Mice (n = 8 per treatment) were inoculated with 30,000 units of S2A, Proleukin, and the same volume of the vehicle twice. PBMCs were isolated 3 days after each inoculation, stained for C45, CD4, CD8, CD25, FoxP3, and CD69, and then the marker distribution was assessed by FACS. (**B**) Representative FACS gating strategy from a mouse treated with S2A. (**C**) FACS data from a combined dataset of 2 independent experiments collected 3 days after 1st inoculation. Data is arrayed, according to lineage (CD4 + and CD8 +) and presented as a percentage of the indicated population on the y-axis. (**D**) Aggregation of FACS data from day 7 (3 days after 2nd inoculation). Data is arranged as in C. (**E**) Plot-box presenting the results of Evans blue assay from murine lung supernatants (n = 5) under the same conditions (Solid line—mean, crushed line—median). (**F**) Representative stained histological cuts of lungs from mice. Error bars represent 95% CI. *, *P* < 0.05; **, *P* < 0.005; ***, *P* < 0.0005.

IL-2 cytokine is a potent inducer of T cells, so next, we assessed T cell induction in vivo. We inoculated mice twice with 30,000 units of S2A, Proleukin, or vehicle control on days 0 and 4, and assessed the phenotype of blood PBMCs on days 3 and 7 (Fig. 3A). Figure 3B shows the representative gating strategy for the flow cytometry analysis. We found that Proleukin and S2A treatments did not cause a substantial change in the CD4^+^ fraction of the CD45^+^ PBMC as compared to the vehicle (Fig. 3C). The CD8^+^ fraction was only slightly induced by S2A treatment (Fig. 3C). Interestingly, the CD4^+^CD25^+^ subset showed significant induction by S2A but not by Proleukin (5.6 fold as compared to vehicle, *P*-value < 0.005, Fig. 3C). This enhancement was even more pronounced on day 7 (*P*-value < 0.0005, Fig. 3D). Notably, the fraction of CD8^+^CD25^+^ subset in the CD45^+^ cells was negligible (Fig. 3C, D). When the classic Treg phenotype was stained (CD4^+^CD25^+^FoxP3^+^ subset within the CD4^+^ cells)^28,29^, the effect of S2A treatment was substantial and significant and became more clear on day 7; while the Proleukin treatment induced negligible amounts of classical Tregs as compared to vehicle (Fig. 3C, D). A 6.1-fold increase in Tregs was observed with S2A treatment on day 7 as compared to vehicle (*P*-value < 0.0005, Fig. 3D). For CD8^+^ cells, the amount of CD25^+^FoxP3^+^ was negligible and had no difference between Proleukin and S2A. Finally, we assessed the CD25^+^CD69^+^ activated T cells^30^. The fraction of the CD25^+^CD69^+^ subsets in the CD4^+^ and CD8^+^ PBMCs of the S2A-treated mice was very low as compared to the considerably enhanced fraction of CD25^+^FoxP3^+^ in the CD4^+^ population (Fig. 3C, D). These data demonstrate that the treatment with S2A, but not with Proleukin, increased the fraction of a prominent subset of the classical Treg phenotype CD4^+^CD25^+^FoxP3^+^ in peripheral blood; this effect was not observed for the CD25^+^CD69^+^ phenotype either in the CD8^+^ or CD4^+^ cells. We also see overall more CD4^+^CD25^+^ CD69^+^FoxP3^+^ than CD4^+^CD25^+^ CD69^+^FoxP3^-^ as a fraction of the CD4^+^ population in S2A treated mice compare to Proleukin treated mice (Fig. S1A), indicating a skew in activation towards T_Regs_.

Mice (n = 5), which were treated with the same protocol described above (twice inoculated with 30,000 units) were also injected intravenously with Evans blue dye prior to sacrifice. The absorbance of the supernatant formamide, in which the excised and washed lungs from the mice were incubated, was measured as an indication of the amount of vascular leakage from the lungs. Lungs from mice treated with S2A showed less leakage compared to Proleukin, which indicated less vascular damage (*P*-value < 0.05, Fig. 3E). In addition histology showed less morphological signs of leakage and tissue damage as can be seen in representative cuts in Fig. 3F.

### S2A inoculation enhances B-16 growth and increases the frequency of Treg within the tumors

**Figure 4:**
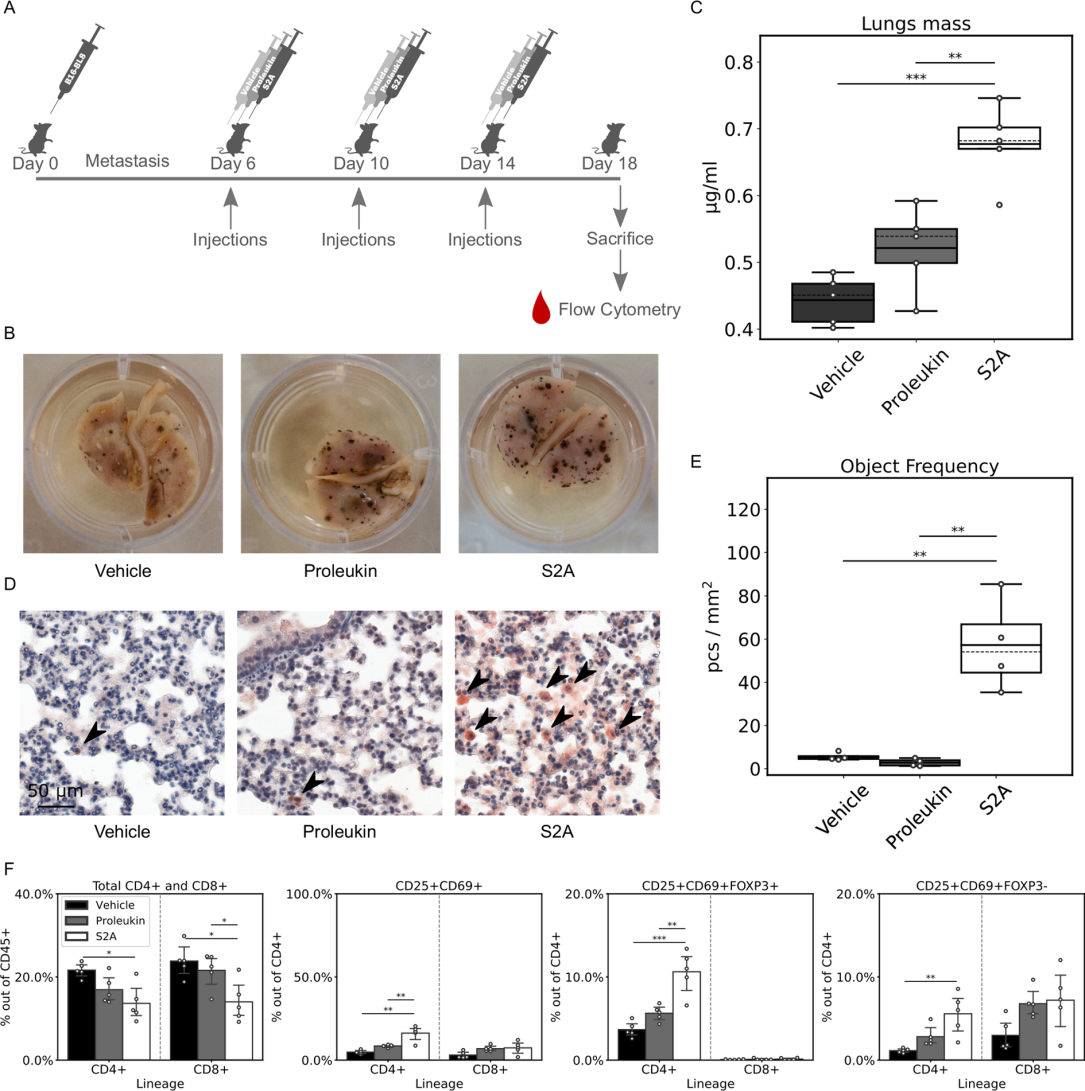
Regulation of B16 growth and Treg frequency in Tumor micro-environment (TME). (**A**) Mice (n = 5 per treatment) were injected with 100,000 B16-BL8 cells i.v. and after 5 days, a session of 3 inoculations, every 4 days was initiated. S2A or Proleukin (30,000 units) were injected at each inoculation. At the 4th day after the last inoculation, mice were sacrificed, and lungs were removed weighed and fixated for immunohistochemistry. (**B**) A representative example of a lung covered with B16 metastasis nodules. (**C**) Lung mass (continuous line—mean, dashed line—median). (**D**) Immunohistochemistry staining of FoxP3 (cells indicated by red arrows) in the representative cut of the metastasized lungs. (**E**) Graph showing the quantitative analysis of FoxP3 immunohistochemical staining in B16-BL8 metastatic lungs. The analysis was performed using Histoquant™ software and expressed in terms of object frequency (pcs/mm^2^). (**F**) FACS analysis of PBMCs isolated at the day of sacrifice arranged according to lineage and presented as percentage of the population noted on the y-axis. Error bars represent 95% CI. *, *P* < 0.05; **, *P* < 0.005; ***, *P* < 0.0005.

T cells can exert strong activities against cancer, while Treg can suppress tumor cell rejection in vivo^31^. Following our initial observations in vivo, we wanted to examine the possible impact of S2A on the immune response to cancer cells. We injected mice (n = 5 per treatment group) with 1 × 10^5^ cells of the melanoma cell-line—B16-BL8^32^ and allowed metastasis to form in the lungs for the next 5 days. Then we treated the mice with 30,000 units of S2A, Proleukin, and the equivalent volume of vehicle as control (Fig. 4A). Four days after the last injection, we examined the lungs, finding melanoma nodules on their surface in all treatment groups (Fig. 4B). In the first assessment for the mass of tumors by weight, we found that the lungs removed from mice treated with S2A were significantly heavier than Proleukin treated or controls (1.3 and 1.5 fold, respectively, *P*-value < 0.005, Fig. 4C). Immunohistological sections of the lungs were made and stained for FoxP3 to evaluate the tumor micro-environment and overall histology of the nodules (Fig. 4D). The S2A group showed a significantly higher frequency of FoxP3^+^ cells (tenfold, *P*-value < 0.005, Fig. 4E). In addition, we phenotyped PBMCs extracted from the mice at the day of sacrifice and found that S2A treated mice showed a significant decrease in CD4^+^ and CD8^+^ subsets of the CD45^+^ population (0.6 and 0.58 folds respectively, *P*-value < 0.005, Fig. 4F) compared to vehicle-treated. On the other hand, the CD4^+^CD25^+^ and CD8^+^CD25^+^ subsets were induced significantly (5.4 and 5.3 fold, *P*-value < 0.05) in S2A compared to vehicle (Fig. 4F). Classical Treg (CD4^+^CD25^+^FoxP3^+^) subset increased significantly (5.3 fold, *P*-value < 0.05, Fig. 4F). Interestingly, the CD8^+^CD25^+^FoxP3^+^ subset also increased significantly (8.2 fold, *P*-value < 0.05, Fig. 4F) even though the absolute percentage of the CD8^+^ population remained lower than the classical Tregs. Induction of CD25^+^CD69^+^ subsets of both CD4^+^ and CD8^+^ populations was negligible (Fig. 4F). These data demonstrate strong immune-modulating activities of the S2A in animals with highly metastatic tumors. The treatment with S2A induced a robust increase of the Treg populations and an increase of metastatic tumor growth.

### S2A ameliorates DSS-induced colitis and improves recovery

**Figure 5:**
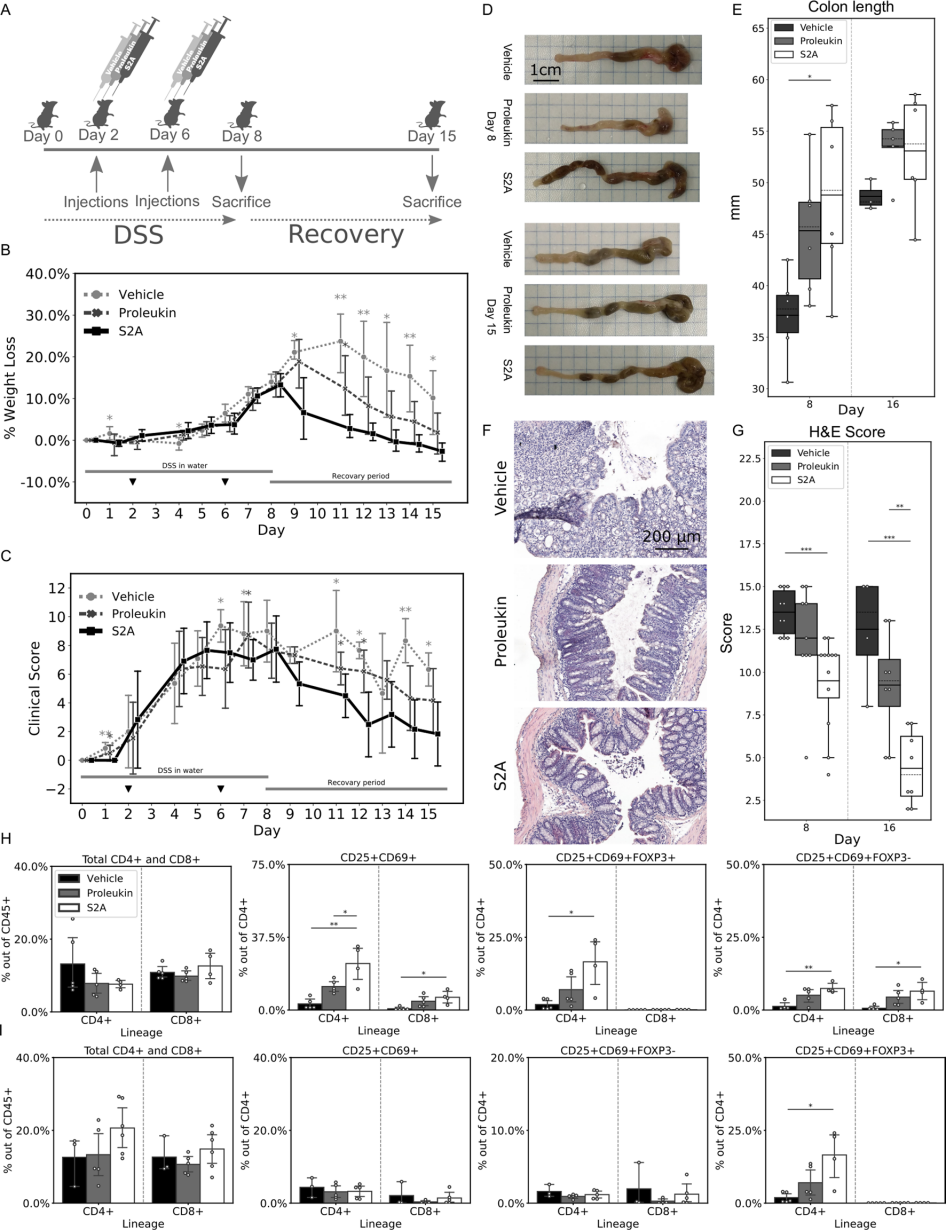
Effects of S2A on DSS induced colitis. (**A**) Mice (n = 12 per treatment) were given water supplemented with DSS, which induces colitis, for a week, during which mice were inoculated twice (day 2 and 6) with 30,000 units of S2A, Proleukin and a corresponding volume of vehicle. After this period, half of the mice were sacrificed (n = 6 per treatment), and half were left to enter a recovery period of a week before sacrifice (n = 6 per treatment). (**B**) Mice were weighed throughout the experiment, and weight loss as a percentage of original weight was plotted against time (black filled down pointing triangle indicates inoculations). Two-way ANOVA was calculated using SPSS, where the combined effect of treatment and time was found to be significant (*P* < 0.0005). Post-hoc test revealed S2A was significantly different from Proleukin (*P* < 0.05) and vehicle (*P* < 0.0005). (**C**) In addition to weight loss, clinical data (diarrhea and anal bleeding) was collected and factored with weight loss to compile the clinical score, which is plotted against time (black filled down pointing triangle indicates inoculations). Two-way ANOVA was carried in SPSS, and the combined effect of treatments and time was found to be significant (*P* < 0.0005). The post-hoc test showed a significant difference between S2A and vehicle but not with Proleukin. However, when considering only the recovery period, statistical significance arises between S2A and Proleukin (*P* < 0.005). (**D**) A representative photograph of colons removed from sacrificed mice, at the end of the DSS period (day 8) and at the end of the recovery period (day 15). (**E**) Colon length was measured the following sacrifice, (continuous line—mean, dashed line—median). (**F**) Representative images of hematoxylin and eosin (H&E) stained colon tissues. (**G**) Quantification of H&E staining. (**H**, **I**) PBMCs were isolated during sacrifice day. FACS analysis is arranged according to lineage and presented as a percentage of the population noted on the y-axis. Error bars represent 95% CI. *, *P* < 0.05; **, *P* < 0.005; ***, *P* < 0.0005.

Following the strong immune-suppressing impact observed above, we next sought to test for a possible beneficial impact of the S2A in autoimmune pathology. We induced acute colitis in mice by Dextran Sodium Sulfate (DSS)^30^. The DSS-induced colitis is resembles Crohn Disease in people. All treated mice were divided in 3 groups (n = 12 per treatment group), which were treated at days 2 and 6 with 30,000 units S2A, or Proleukin, or with the equivalent volume of vehicle, as illustrated in Fig. 5A. All mice were evaluated for clinical signs of colitis (diarrhea and anal bleeding) and were also weighed every day after DSS administration (Fig. 5B, C). Weight loss, as a percentage of the initial weight, showed a significant difference in a two-way ANOVA test (*P*-value < 0.0005), The effect of treatment alone was also significant (*P*-value < 0.0005), and in post-hoc tests, weight loss in the S2A treated group was found to be significantly less than those in the Proleukin (*P*-value < 0.05) and the vehicle (*P*-value < 0.0005) groups. Clinical signs were factored with weight loss to formulate a clinical score (Fig. 5C). In a two-way ANOVA, a significant difference in the clinical score (*P*-value < 0.0005) attributed to the combined effect of treatment and time was found, as well as a significant difference due only to the component of treatment (*P*-value < 0.0005). In a post-hoc test, a significant difference in the clinical score was found between the S2A group and the vehicle group (*P*-value < 0.05), but not between S2A and Proleukin. Nonetheless, when considering only the recovery period a statistical difference between the S2A group and the Proleukin group is evident (*P*-value < 0.005).

At the end of the DSS period, half of the mice in each group (n = 6 per treatment group) were sacrificed, and their colons were removed and measured for their length. The remaining mice were sacrificed at day 15, and their colons were also evaluated (Fig. 5D). Colon length of the S2A group was significantly longer at the end of the DSS period as compared to the vehicle group (*P*-value < 0.05), and longer (but not statistically significant) as compared to the Proleukin group. In a recovery period, the significance was abolished, although the average colon is still longer in the S2A group in comparison to the vehicle group (Fig. 5E).

Colons were cut and stained with H&E for histological analysis. Damage to the tissue and crypts, as well as immune cell involvement were assessed and scored (Fig. 5F). At the end of the DSS treatment, S2A treated mice faired significantly better than the vehicle mice (1.4-fold better, *P*-value < 0.0005), and better than Proleukin treated mice although not statistically significant (Fig. 5G). At day 15, S2A treated mice faired significantly better than mice treated with vehicle (2.8-fold better, *P*-value < 0.0005) or Proleukin (2.1-fold better, *P*-value < 0.005, Fig. 5G). We observed less recruitment of immune cells and better colon epithelial cell repair in mice treated with S2A.

PBMC populations were analyzed at the end of the DSS treatment and also in the recovery period (day 15). While no relative induction of the CD4^+^ subpopulation of the CD45^+^ population was apparent at the end of the DSS period (day 8), and the CD4^+^CD25^+^ sub-populations were almost negligible as a percentage of the CD45^+^ population in all groups, the CD4^+^CD25^+^FoxP3^+^ (classical Treg) subset of the CD4^+^ population was significantly induced in the S2A treated mice as compared to both Proleukin and vehicle treated mice (2.7 folds as compared to vehicle, *P*-value < 0.0005, and 1.3 folds compared to Proleukin, *P*-value < 0.05). CD8^+^CD25^+^FoxP3^+^ subset was somewhat induced in the S2A group but not significantly at the end of the DSS administration (Fig. 5H). No difference was observed in both CD4^+^CD25^+^CD69^+^ or CD8^+^CD25^+^CD69^+^ populations was measured at the end of the DSS treatment. We did not find differences in other population under different treatments no at day 10, neither at day 15(Fig. 5I).

### S2A can delay rheumatoid arthritis (RA) progression

**Figure 6:**
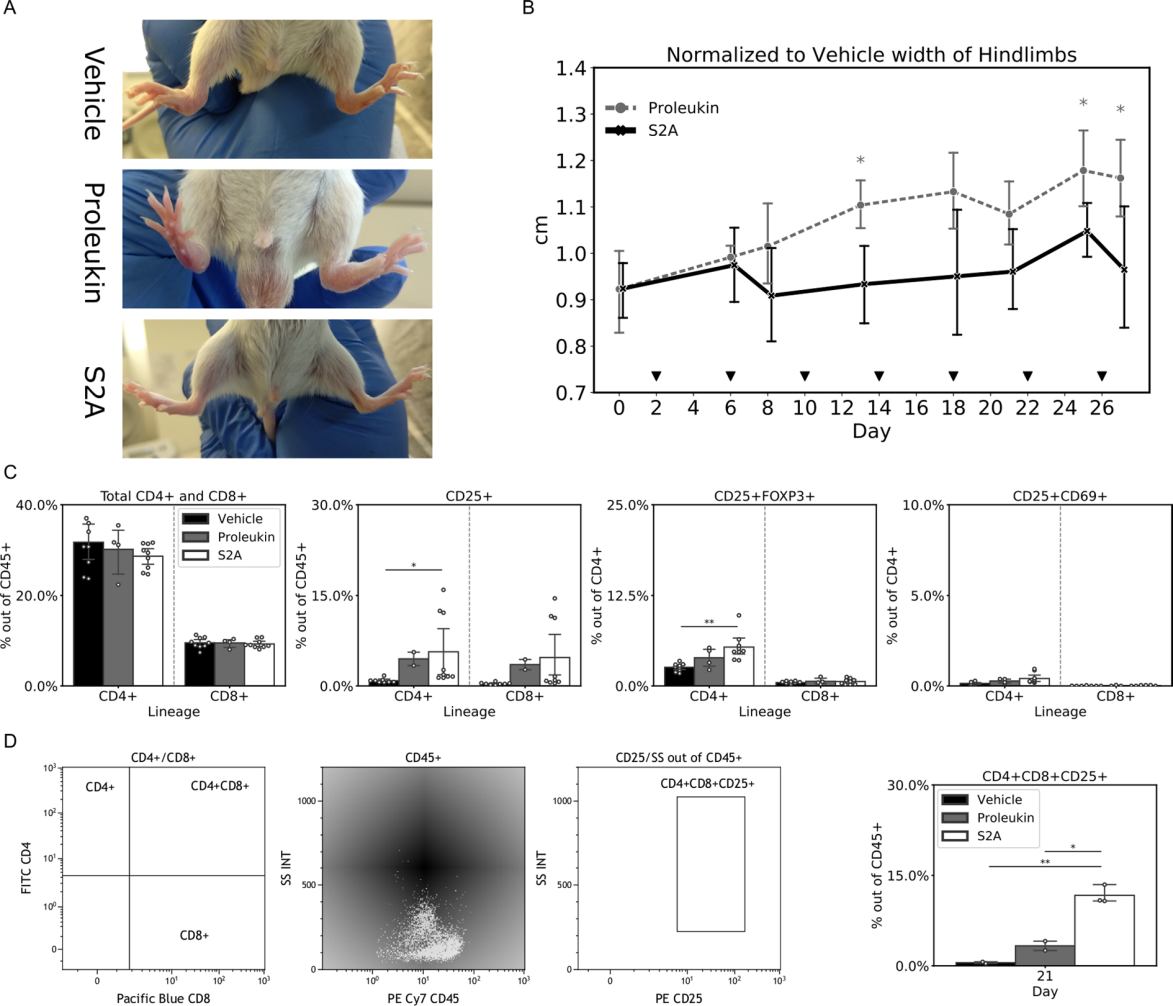
S2A's effect on the development of rheumatoid arthritis (RA) in the IL-1ra KO model. IL-1ra KO mice spontaneously develop RA at the age of 3–6 weeks. Mice ((n = 8 in the S2A group, n = 4 in the Proleukin group and n = 9 in the vehicle group) were inoculated with 100,000 units every 4th days for a total of 7 inoculations. (**A**) Representative examples for the RA phenotype (inflamed and swollen hind-limb joints). (**B**) Joints width was measured, normalized to the vehicle, and plotted against time (black filled down pointing triangle indicates inoculations). Two-way ANOVA was performed on data, and while combined effect was not statistically significant, the treatment component was significant (*P* < 0.0005). (**C**) At day 21, PBMCs were isolated. FACS analysis is arranged according to lineage and presented as a percentage of the population noted on the y-axis. (**D**) In addition to the normal phenotypes, this model presented a highly granulated double-positive (CD4 + CD8 +) phenotype, which also seemed to be almost entirely positive to CD25. Presented is a FACS gating scheme of this population (**E**) Representative plot of the double-positive phenotype induction in mice treated with Proleukin S2A and vehicle. Error bars represent 95% CI. *, *P* < 0.05; ** *P* < 0.005; ***, *P* < 0.0005.

With the above data from xenogenic cancer and induced colitis models, we next examined the ability of S2A to treat an endogenous autoimmunity disease. IL-1 receptor antagonist knockout (IL-1ra KO) mice spontaneously develop rheumatoid arthritis (RA) 3–6 weeks after birth, as a result of the inability to counterbalance the pro-inflammatory effect of IL-1^33^. We sought to examine whether S2A may attenuate the severity and progression of RA compared to Proleukin. Mice at the age of 4 weeks (n = 8 in the S2A group, n = 4 in the Proleukin group and n = 9 in the vehicle group) were inoculated with 100,000 units of S2A, Proleukin, or Vehicle control. Inoculation began at day 2, and performed every 4th day after that, for a total of 7 inoculations. As expected, signs of RA included apparent inflammation and swelling of the hind-limb joints (Fig. 6A). Joints’ widths were measured throughout the experiment, and two-way ANOVA performed on the data normalized (Fig. 6B). While the combined effect of time and treatment was not significant, the treatment component was significant (*P*-value < 0.0005). PBMCs were FACS analyzed on day 21 (3 days after 5th inoculation). Significant induction of CD4^+^CD25^+^ subset of CD45^+^ in the S2A group was measured on day 21 compared to the vehicle group (6.4 folds, *P*-value < 0.05). In addition, a small induction was also observed in the Proleukin group (1.8 folds, not statistically significant). The classical Treg phenotype (CD4^+^CD25^+^FoxP3^+^), showed significant induction in the S2A group, compared to the vehicle group (2.1 folds, *P*-value < 0.005). CD8^+^CD25^+^FoxP3^+^ measured levels are negligible in all treatment groups (Fig. 6C). Intriguingly, a population of cells that express a double positive phenotype (CD4^+^CD8^+^) was prevalent in this model (Fig. 6D). These cells were present regardless of treatment; they were mostly CD25 positive and highly granulated (high side-scatter values) (Fig. 6D). The S2A treatment further increased the frequencies of this population in blood, with 21 folds more (*P*-value < 0.0005, Fig. 6E).

## Discussion

The past decade has seen a renaissance in IL-2 related research. Much of this research is focused on new strategies to re-introduce IL-2 into the clinics^8,34–37^. One approach is treating with low doses of IL-2 that preferentially induce Treg based on the abundance in Treg of the high-affinity complex of the IL-2 receptor ^2,38^. This kind of treatment achieved results in ameliorating auto-immune symptoms of different syndromes. The low-dose approach also means fewer side-effects; however, this strategy still suffers from the fact that IL-2 has short serum half-life. Some research was also done on this topic^2^. Gillies et al*.* significantly prolonged IL-2 half-life by ligating the protein to an Fc fragment, or even a whole antibody^39^. Others approached the IL-2 conundrum by the introduction of point-mutations to the protein binding sites, thus changing its affinity to the different components of the IL-2 receptor. Levin et al*.* described certain point mutations that decrease affinity to IL2Rα as well as others that increase affinity to IL2Rβ, and subsequently might induce a stronger CD8^+^ and NK response^10^. Other mutations increase affinity to IL2Rα, which might lend this recombinant IL-2 the ability to induce a strong Treg response^10^.

Our approach was initially conceived as a solution to the half-life problem. It involved the ligation of two highly glycosylated flanking sequences to IL-2 in hopes that these will stabilize the protein in the serum and will enable a slow release from the injection site. A slow-releasing stable IL-2 would make treatment easier for patients and more for clinicians. Evidently, we show that our S2A has significantly longer half-life and higher bioavailability than Proleukin, which is used in the clinics. It is possible that the mere size of the glycosylated flanks slows down the diffusion of S2A from the tissue to the bloodstream, effectively acting as a pump mechanism^40,41^. In the serum itself, S2A is probably more resistant to proteases and clears at a much slower rate through the renal pathway, allowing its effect to last longer and permeate throughout the body, as was suggested for the life-extended GH (Growth Hormone)^19,42^.

It also seems S2A has a better ability to induce proliferation in CTLL-2 assay in vitro, which indicates a more potent agent, either due to better binding to receptors on CTLL-2 cells or due to better stability. It is possible to presume a change to the binding affinity of one or all the different parts of the receptor due to structural interference. Such bias, as well as changes in the constant level of S2A in the sera due to the pump mechanism, can modulate the immune system in many ways, either along a pro-inflammatory vector or anti-inflammatory vector.

Our in vivo experiments show a strong bias towards an anti-inflammatory vector. A sharp increase in the classical Treg population (CD4^+^CD25^+^FoxP3^+^), while retaining a negligible amount of CD8^+^ effector cells after administration, indicates suppression of the system in a naive model. This event could be the result of inducible Tregs (iTregs) induced by S2A, which then suppresses the system by secretion of anti-inflammatory cytokines. iTregs have been shown to play a part in the suppression of auto-immune inflammatory diseases and implicated in the elimination and fatigue of cancer-fighting effector T cells and NK cells in tumor environment^31,43^.

As can be inferred from our melanoma model, it does seem that S2A fails to induce an anti-cancerous reaction, when compared to Proleukin. Lung mass was higher in mice treated with S2A, indicating the formation of more metastasis in the lungs. While in our model Proleukin also failed to exhibit anti-cancerous reaction, it faired better than S2A. Immunohistochemistry of the tumor tissue seems to corroborate the Treg hypothesis, showing much higher infiltration of FoxP3^+^ positive cells to the tumor tissue of S2A treated mice. Peripherally, it seems that Treg levels are lower than in the naive model, which may be attributed either to the pro-inflammatory reaction to the tumor, countering the anti-inflammatory reaction induced by S2A, or rather due to migration of said peripheral Tregs from the blood to the tumor.

Our experiments using auto-immune models, align well with the idea that S2A drives an anti-inflammatory reaction. In the case of rheumatoid arthritis, we demonstrated that S2A treated mice deteriorated slower than Proleukin treated mice. The Treg population in the PBMCs also preserved its difference between the treatment groups, and again it was relatively less pronounced than in the naive experiment—presumably due to migration into inflamed areas or towards strong systemic pro-inflammatory signals. An interesting observation was made in this model regarding a rather large sub-population of peripheral (i.e., not in the thymus) double positive CD4^+^CD8^+^ cells, which was significantly bigger in S2A treated mice. This sub-population only appeared in this model and maybe a consequence of the missing receptor antagonist. Nonetheless, this sub-population was also observed by other research groups in other models and was implicated in the suppression of auto-immune symptoms^44^.

The same trends are clearly visible in the colitis model to even a greater degree, where mice treated with S2A lost weight slower during the disease phase and recovered much faster during the recovery phase. Immunohistochemistry suggests the colons of mice treated by S2A showed fewer signs of inflammation, as also indicated by the length of colons. Treg population in PBMCs is again lower relatively to naive experiment, and by the 16th day (Day15), it is not different in all treatments, due to elapsed time since the last injection. While results for the acute phase of the inflammation were indeed inconclusive, the recovery of S2A treated mice was rapid in compare to Proleukin treated mice. When considering the clinical aspects of cytokine administration, this property might be useful in reducing the recovering time from flare-ups of a plethora of auto-immune inflammatory syndromes, which might have long recovering periods.

Our approach, therefore, successfully achieved a substantial prolongation of IL-2 half-life, with the added effect of promoting an anti-inflammatory reaction. Our method of protein design and production is relatively simple, scalable, and adaptable. Proteins are synthesized as a single unit, which gains most of its mass from post-translational modifications, which entails an easier cloning process. This also enables the easy introduction of modifications and mutations to the sequence. For example, single point mutations to the IL-2 sequence to enhance specific binding to IL2Rβ, creating a bias for anti-cancerous response, rather than anti-inflammatory response. The flanking sequences could be ligated to other endocrine or paracrine signal proteins that up until now were kept out of the clinic due to their short half-life but harbor great potential as treatments for various conditions.

## Materials and methods

### Plasmid design

Protein sequences were designed and sent to Hylabs, where a humanized DNA sequence was matched, and the cassette was inserted into a pCDNA-3.1( +) backbone. Sequence of the protein included an IL-2 signal peptide (MYRMQLLSCIALSLALVTNS), followed by a fragment of the NCR2 (Nkp-44) protein (34AA; residues 130 to 163), the complete sequence of IL-2 (133AA; residues 21 to 153) and a second NCR2 fragment (36AA; residues 164 to 199)^45^. The sequence ends with an HRV 3C cleavage site followed by a 6XHis tag tail. Plasmids were transformed into DH1α bacteria and extracted after proliferation.

### Protein production and purification

Plasmids were transfected into HEK293F cells, which were cultured in shaking flasks. After 3 days, the supernatant was separated from cells using centrifugation and filtering. FPLC process was done using ÄKTA (GE healthcare), where supernatant was supplemented with 500 mM imidazole (Sigma-Aldrich) solution in a ratio of 1:25 and loaded on a nickel-ion column (HisTrap GE healthcare). Elution performed with 500 mM imidazole solution. The washed fraction was concentrated and further cleaned in Amicon (Millipore) with 10 kDa cutoff. Sample concentration was measured using NanoDrop (ThermoFisher Scientific), assuming the extinction coefficient calculated with ExPASy Protparam tool, according to protein sequence. Protein was then diluted to a level of 1 mg/ml, divided into small aliquots in Eppendorf Protein LoBind tubes, and frozen at − 80 °C.

### SDS-PAGE

Five µg of protein solution was denaturized in β-mercaptoethanol and SDS solution at 95 °C for 5 min, before loading on SDS-PAGE 12% acrylamide gel, and ran at 100 V. Protein was stained using InstantBlue (expedeon).

### ELISA for binding assay

We used the Biolegend ELISA MAX kit for the detection of human IL-2. For binding assay, purified S2A was compared to recombinant human IL-2, Aldesleukin (Peprotech, Cat no. 200–02). 96-well ELISA plates were coated with capture ab according to the protocol supplied with the kit, and then proteins were serially-diluted in the plate. Capture ab, HRP, and TMB were added according to the protocol supplied with the kit. Plates were read sequentially, at 650 nm without stop solution, and the best reading was chosen. Data were fitted to 4 parameters logistic (4PL) model, and EC_50_ was extrapolated, using GraphPad Prism 5.0.

### MTT assay using CTLL-2 cells for specific activity evaluation

CTLL-2 cells were obtained with the gracious help of Drs. Esther Tzehoval and Lea Eisenbach from the Weizmann Institute. Cells were cultured in complete RPMI supplemented with 25 units/ml of recombinant human IL-2 (Proleukin Novartis Pharma GmbH, Germany). In 96-well plates, serial dilutions of cytokines were carried out. CTLL-2 cells were then washed three times with PBS, and 1 × 10^4^ cells were seeded per well in a 96 well plate, in a volume of media equal to the volume of media in the well, to a total of 90µL, essentially halving the concentration of cytokines across all wells. Cells were incubated for 2 days, and on the third, 10 µL 5 mg/ml MTT (Sigma-Aldrich) solution was added to each well. Cells were further incubated to allow metabolism of the MTT, and after 1–2 h, formazan crystals were dissolved by adding 100µL per well of isopropanol (supplemented with 0.5% HCl) and pipetting. Plates were read at 570 nm, and 630 nm and results subtracted (630 nm from 570 nm) to obtain the normalized results, which were fitted to 4 parameters logistic (4PL) model and ED_50_ was extrapolated, using GraphPad Prism 5.0. Specific activity is calculated according to the formula:

$$Specific\;Activity \left( {\text{Units/mg}} \right) = \frac{{1 \times 10^{6} }}{{ED_{50} }} \left( {\text{ng/mL}} \right)$$

### Preparation of cytokines for injection

The number of units available for each cytokine was determined according to CTLL-2 assay. Prior to the experiment, aliquots of cytokines were made with the required amount of units to be injected, taking into consideration a 28% loss due to two defrost cycles. The volume and dilution of aliquots were calculated to accommodate a 100 µL injection volume per mouse. Dilution was made in sterile PBS 1X solution (Hy-labs). Aliquots were then refrozen at − 80 °C until injection time. The vehicle is PBS 1X solution.

### Pharmacokinetics

Mice (5 mice per treatment) received subcutaneous (SC) injection of Proleukin (180 pmol, 8000 units) and S2A (15 pmol, 30 pmol, and 60 pmol, that correspond to 3000 units, 6000 units and 12,000 units, respectively) at time 0. Blood samples (10 µL) were collected from the tail vein prior to the dosing, at 10 and 30 min, then at 1, 2, 4, 24, 30, 42, and 72 h. Alsever’s solution (200 µL) was added to the samples to prevent coagulation, and they were stored at -80 °C pending analysis. Blood concentrations of Proleukin and S2A were measured using the Biolegend ELISA MAX kit for the detection of human IL-2 based on appropriate calibration curves. Non-compartmental pharmacokinetic analysis of the observed concentration versus time data was performed using the PKSolver 2.0 add-in for Microsoft Excel^46^ for each mouse separately and averaged by treatment post-analysis.

### PBMCs isolation and FACS analysis

200 µL blood was collected into a tube coated with EDTA to prevent clotting and immediately added to 1 ml Alsever’s solution. RBCs were lysed in ACK buffer made in the lab. WBCs were counted, and 100,000 cells of each sample were transferred into a well of a 96-well plate where the subsequent procedure was done. Cells were blocked with anti-CD16/CD32 (Biolegend, clone: 93), and membranal proteins were stained with anti-CD45 PE/Cy7 (Biolegend, clone: 30-F11), anti-CD4 FITC (Biolegend, clone: GK1.5), anti-CD8 Pacific Blue (Biolegend, clone: 53–6.7), anti-CD25 PE (Biolegend, clone: PC61), and anti-CD69 APC/Cy7 (Biolegend, clone: H1.2F3). Cells were then fixed and permeabilized using the TruNuclear kit (Biolegend) and stained with anti-FoxP3 APC (ThermoFisher Scientific, clone: FJK-16 s). Cells were than read in Beckman Coulter Gallios flow cytometer. Results were gated and analyzed using Kaluza 2.1.

### Immunohistochemistry

Tissue sections of 5 μm thickness were cut from FFPE blocks using a fully automated rotary microtome (Leica RM2255). Morphology of tissues was assessed using staining with hematoxylin (Mayer) and 1% alcoholic eosin (Pioneer research chemical Ltd, UK) staining. For IHC staining, tissue sections were serially deparaffinized with xylene (3 times for 5 min) and then rehydrated with absolute ethanol (2 times for 5 min). The endogenous peroxidase activity of sections was quenched with methanolic 3% H_2_O_2_ solutions for 20 min. After washing with doubled distilled water, antigen retrieval was done at 95 °C using citrate buffer pH 6.0 (Zytomed) as an antigen unmasking solution. Sections were then washed, and ImmPRESS universal reagent (Vector Laboratories, MP-7500) was used according to the manufacturer's protocol for the blocking. After the blocking, sections were incubated overnight at 4 °C with primary antibody against FoxP3 (diluted 1:50, BioLegend, cat no—320,001). Following extensive washings, sections were then incubated with HRP conjugated secondary antibody from ImmPRESS universal reagent (Vector Laboratories, MP-7500) for 30 min. Lastly, staining was visualized by using AEC solution (Zymed Laboratories, San Francisco, CA, USA) according to manufacturer’s protocol, the reaction stopped in the water, counterstained with hematoxylin and tissue sections were mounted with aqueous mounting medium (Vectamount™ AQ, cat no–H-5501).

Tissue images were scanned by a panoramic scanner and analyzed by HistoQuantTM software (3D Histech). For FoxP3 staining, the software calculated the number of positive nuclei and the annotated area for each tissue, and the value was expressed as object frequency (pcs/mm^2^).

### Naive model

C57bl/6 WT mice, 8 weeks old were obtained from Envigo and injected with 30,000 IU of Proleukin (n = 8), S2A (n = 8) and the same volume of PBS (n = 8) at day 0. At day 3 blood was drawn and PBMCs isolated for flow-cytometry (as described in the “PBMCs isolation and FACS analysis” subsection). A day later (day 4) mice were injected again in the same manner. At day 7, mice were sacrificed and blood was drawn and PBMCs isolated for flow-cytometry.

### Evans blue

On the day of sacrifice in the in vivo naive model for T cell induction, mice were injected intravenously with 100 µl 2% Evans blue solution freshly prepared in sterile PBS. The mice were allowed to recuperate and remain mobile for two h, after which, lungs were harvested, washed twice in PBS, and placed into tubes containing 2 ml Formamide and incubated at 37 °C for 24 h. After incubation, absorbance of the supernatant was measured at 650 nm and compared against Evans blue standard curve^47^.

### Metastatic melanoma model

B16-BL8 cells were obtained with the gracious help of Dr. Noam Levaot of Ben-Gurion University in the Negev. Cells were cultured in complete RPMI. 8 weeks old C57bl/6 WT mice were obtained from Envigo and acclimatized before experimentation (n = 5 per treatment group). Before injection, cells were washed and suspended in serum-free RPMI medium. At day 0, each mouse was inoculated intravenously in the tail vein with 1 × 10^5^ cells (in 100 µl media without any supplement). Mice were allowed 5 days for metastasis to develop in lungs before cytokine injection. Each mouse was injected with 30,000 units of respective cytokine dissolved in PBS 1X in a total volume of 100 µl per injection as described under “Preparation of cytokines for injection” section heading. The vehicle was PBSx1. Injections were given subcutaneously every 4th day after 5 days of B16-BL8 inoculation. Blood samples were collected on day 18 (sacrifice). PBMC was stained and analyzed, as described above.‏ On day 18, mice were sacrificed, lungs were harvested and weighed. Immunohistochemistry was performed on excised lungs, as described in the “Immunohistochemistry” section part.

### Colitis model

To induce colitis, 6 weeks old C57bl/6 WT mice (n = 12 per treatment group) obtained from Envigo were given 2.5% Dextran Sulfate Sodium (DSS, TdB) in the drinking water for 1 week (defined as DSS period). During this period, mice were injected twice with cytokines on day 2 and 6, counted from the onset of DSS administration. On day 8, half of the mice in each treatment group (n = 6) were sacrificed. Blood was drawn and colon removed and measured for length. Samples from colons were fixed for IHC. The rest of the mice (n = 6 per treatment group) were allowed 1 week of recovery (plain water without DSS was given) before sacrificed in an identical manner on day 15. Mice were weighed every day throughout the experiment. Clinical manifestation of diarrhea and blood in stool were also tracked by touching a tissue paper to the mouse anus and examining the stain. Clinical scoring was done by factoring weight loss percentage with clinical manifestation (weight: 1–5% loss—1 point, 6–10% loss—2 points, 11–20% loss—3 points, more than 20%—4 points. Stool consistency: well formed—0 points, semi formed stool—2 points, liquid stool—4 points). Each mouse was injected with 30,000 units of respective cytokine dissolved in PBS 1X in a total volume of 100 µL per injection, as described in "Preparation of cytokines for injection" section part. The vehicle was PBS 1X. PBMCs were stained and analyzed, as described above. IHC was scored by summing the parameters—depth of injury (none—0 points, mucosal and submucosal—2 points, transmural—3 points), crypt damage (none—0 points, basal one-third damaged—1 point, basal two-thirds damaged—2 points, only surface epithelium intact—3 points, entire crypt and epithelium lost—4 points), percentage of tissue involvement (1–25%—1 point, 26–50%—2 points, 51–75%—3 points, 76–100%—4 points), percentage of immune cell infiltration (1–25%—1 point, 26–50%—2 points, 51–75%—3 points, 76–100%—4 points).

### Rheumatoid arthritis model

These mice spontaneously develop rheumatoid arthritis as they age. For this experiment, mice were chosen before showing clinical signs, at the age of 3 weeks. Hind limb joints were then measured using a digital caliper, twice a week. Each mouse was injected with 100,000 units of respective cytokine dissolved in PBS in a total volume of 100 µL per injection as described in “Preparation of cytokines for injection” section part, with PBS 1X as the vehicle (n = 8 in the S2A group, n = 4 in the Proleukin group and n = 9 in the vehicle group). Injections were given every 4 days, for a total of 7 doses. On the 21st day, blood was sampled, and PBMCs were stained and analyzed as described above.

### Statistics

Student's t-tests and Mann–Whitney U tests were performed using a python script utilizing the statistics module of the ‘SciPy' library^48^. Means, medians, confidence intervals, and folds were calculated using the 'pandas' library^49^. ANOVAs were calculated in Statistica.

### Ethical statement regarding experiments with mice

All animal procedures were approved by the Ben-Gurion University of the Negev's committee for the ethical care and use of animals in experiments (Approval number: IL-17-05-2017) and the experiments were performed in accordance with the guiding rules of The Ministry of Health and the Council for Experiments of Animal Subjects. All animal models were designed and implemented according to the ARRIVE guidelines^50^.

## Supplementary Information

Supplementary Information 1.
