# Supplementary material for: Life-extended glycosylated IL-2 promotes Treg induction and suppression of autoimmunity
Source: Sci Rep. 2021 Apr 7;11:7676. doi: 10.1038/s41598-021-87102-4 (PMC8027413; doi:10.1038/s41598-021-87102-4)
Supplement: Supplementary file 1 — Supplementary Information 1. [file 41598_2021_87102_MOESM1_ESM.docx]

**Life-Extended Glycosylated IL-2 Promotes Treg Induction and Suppression of Autoimmunity**

Aner Ottolenghi^1,2,*^, Priyanka Bolel^1,2,*^, Rhitajit Sarkar^1^, Yariv Greenshpan^1,2^, Muhammed Iraqi^1,2^, Susmita Ghosh^1,2^, Baisali Bhattacharya^1^, Zoe V. Taylor^1^, Kiran Kundu^1,2^, Olga Radinsky^1^, Roi Gazit^1,2^, David Stepensky^3^, Ron N. Apte^1^, Elena Voronov^1^, Angel Porgador^1,2,#^

^1^ Faculty of Health Sciences, The Shraga Segal Department of Microbiology, Immunology, and Genetics, Ben-Gurion University of the Negev, Beer Sheva, 84105, Israel.

^2^ National Institute for Biotechnology in the Negev, Ben-Gurion University of the Negev, Beer Sheva, 84105, Israel.

^3^ Faculty of Health Sciences, Department of Clinical Biochemistry and Pharmacology, Ben-Gurion University of the Negev, Beer Sheva, 84105, Israel.

*These authors have contributed equally to this work.

^#^ corresponding author:

Angel Angel Porgador, the Shraga Segal Department of Microbiology, Immunology, and Genetic, Faculty of Health Sciences, Ben-Gurion University of the Negev, Beer Sheva, Israel

Phone: 972-8-6477283, Fax: 972-8-6477626, Email: [angel@bgu.ac.il](mailto:angel@bgu.ac.il)

**Supplementary data:**

Figure S1:


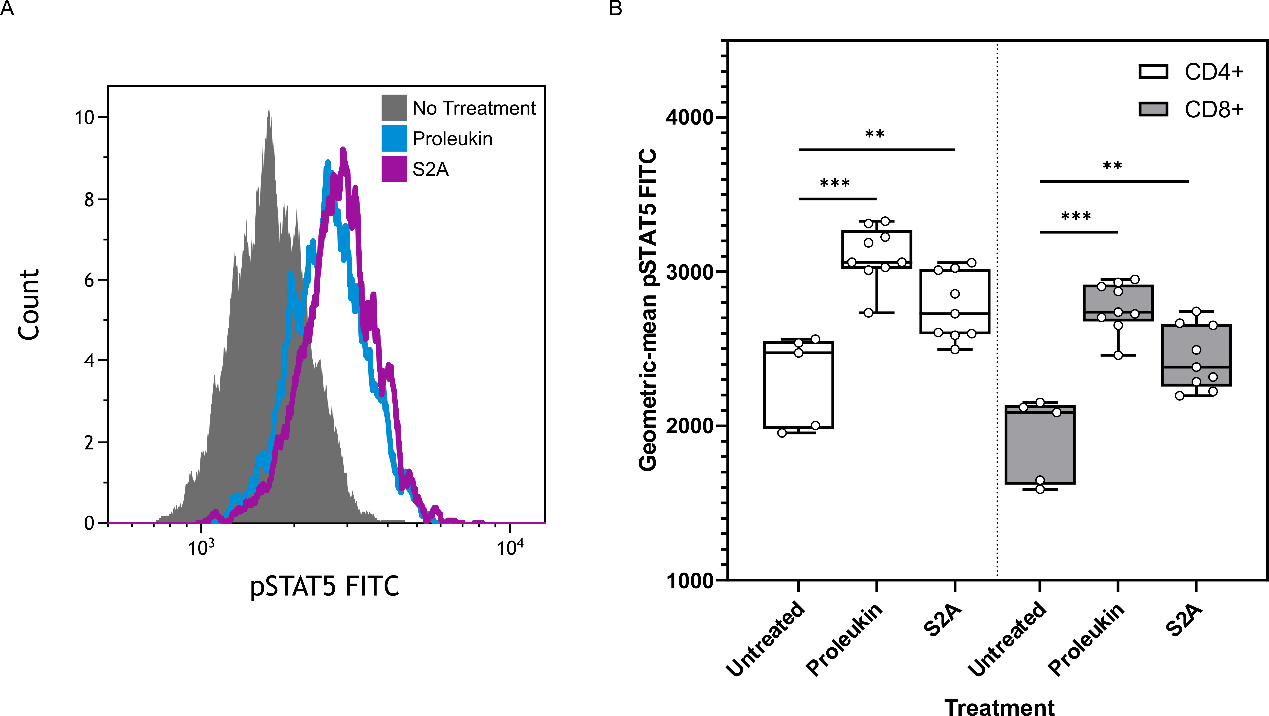


**Figure S1: Activation of the pSTAT5 pathway in human T-cell:** Human T-cells activated by OKT3 anti-body, were incubated in media without IL-2 supplementation for a night. Cells exposed to cytokines for 10 minutes and immediately fixated in PFA, and stained for CD4, CD8 and pSTAT5. Samples were then evaluated for STAT5 phosphorylation using flow cytometry. (A) A representative histogram of CD4^+^ population exhibiting a shift in the distribution. (B) Summation of the flow cytometry results (n=9 repetitions). Error bars represent 95% CI. *, P < 0.05; ** P < 0.005; ***, P <0.0005.

Figure S2:


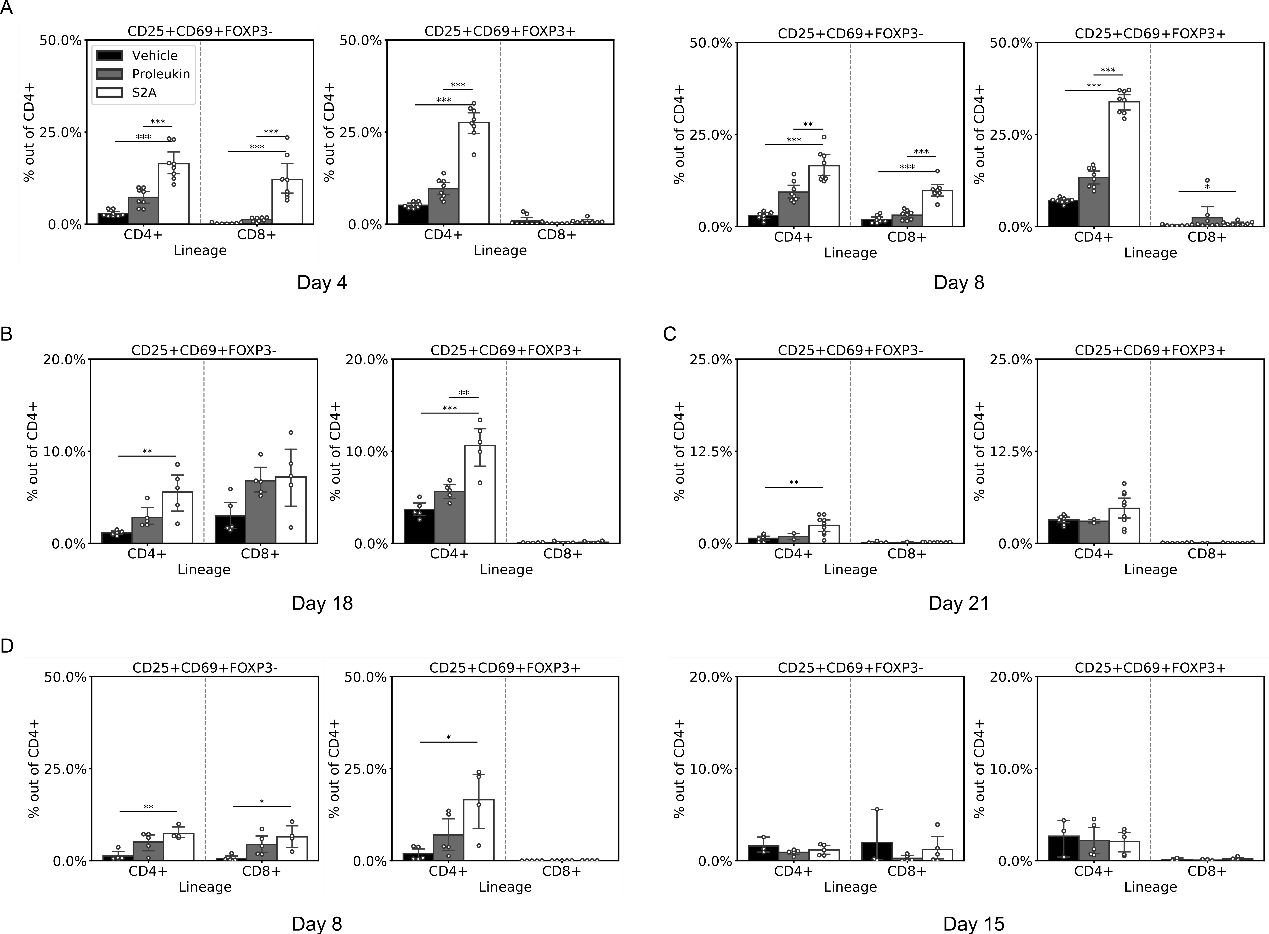


**Figure S2: Re-analysis of flow cytometry data, highlighting FoxP3^+^ and FoxP3^-^ population in the activated subset:** (A) Data from naive mice model (n=8 per treatment). (B) Data from B16 melanoma inoculation model (n=5 per treatment). (C) Data from rheumatoid arthritis (RA) IL-1ra KO model (n=5-9 per treatment). (D) Data from DSS induced colitis model (n=6 per treatment in every time point, n=12 per treatment overall). Error bars represent 95% CI. *, P < 0.05; ** P < 0.005; ***, P <0.0005.

Figure S3:


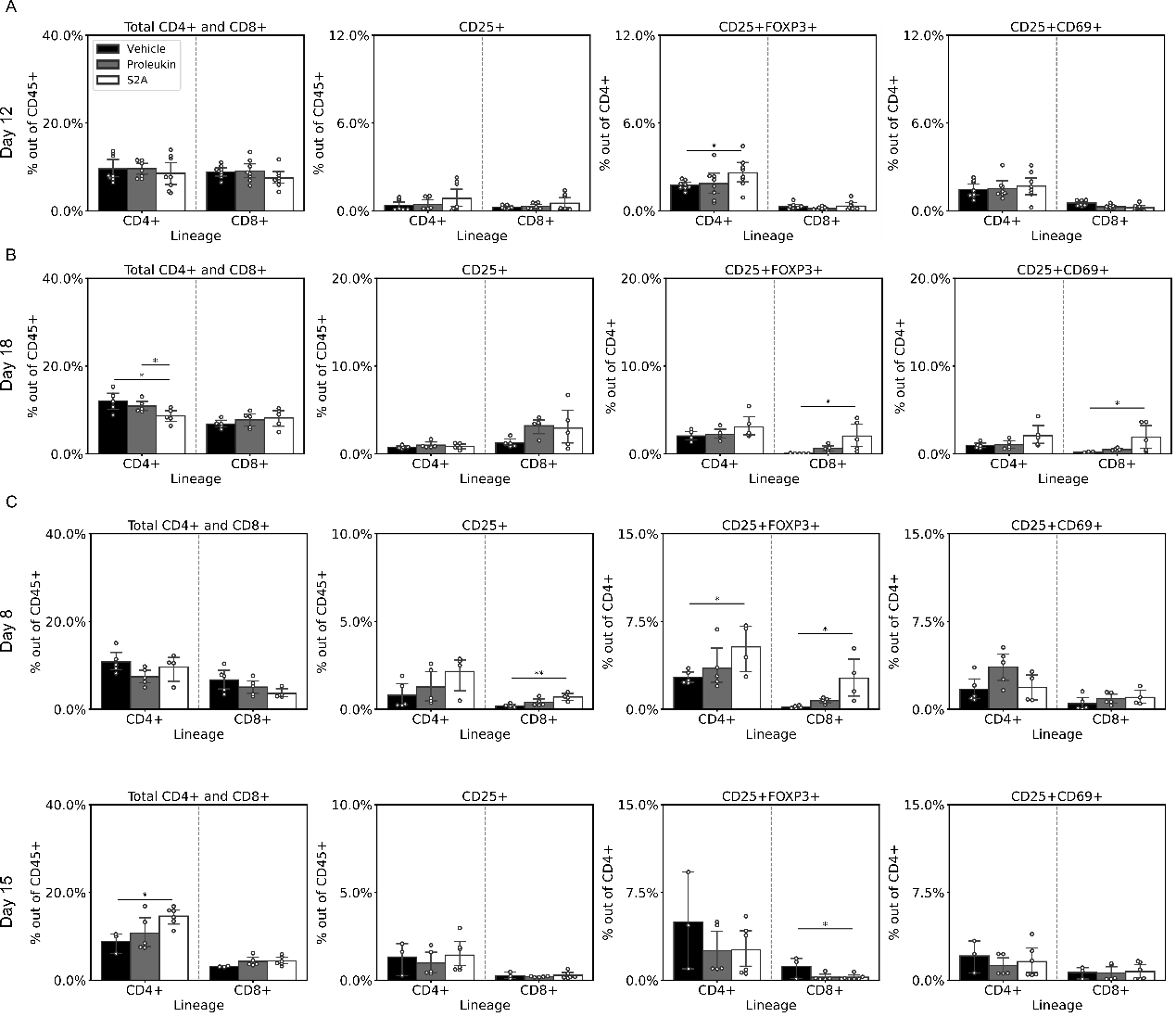


**Figure S3: Flow cytometry data from spleen**: (A) Data from naive mice model (n=8 per treatment). Spleens were harvested at day 12. (B) Data from B16 melanoma inoculation model (n=5 per treatment). Spleens were harvested at day 18. (C) Data from DSS induced colitis model (n=6 per treatment in every time point, n=12 per treatment overall). Spleens were harvested at days 8 and 15. Error bars represent 95% CI. *, P < 0.05; ** P < 0.005; ***, P <0.0005.

Figure S4:


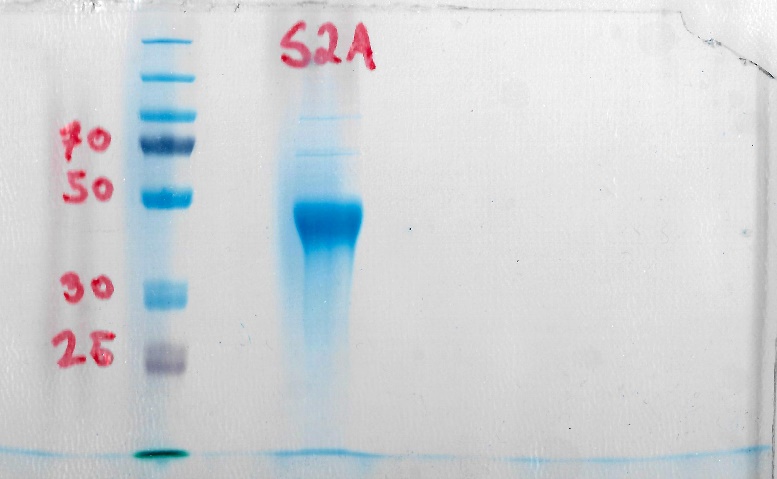


**Figure S4: SDS-PAGE of S2A**: A raw uncropped image of the SDS-PAGE 10% gel of S2A.
